# Large Language Models in Oncology: Revolution or Cause for Concern?

**DOI:** 10.3390/curroncol31040137

**Published:** 2024-03-29

**Authors:** Aydin Caglayan, Wojciech Slusarczyk, Rukhshana Dina Rabbani, Aruni Ghose, Vasileios Papadopoulos, Stergios Boussios

**Affiliations:** 1 Department of Medical Oncology, Medway NHS Foundation Trust, Gillingham ME7 5NY, UK; aydin.caglayan@nhs.net (A.C.); rukhshana.rabbani@nhs.net (R.D.R.); aruni.ghose1@gmail.com (A.G.); 2 Kent Medway Medical School, University of Kent, Canterbury CT2 7LX, UK; wojciech.slusarczyk@nhs.net; 3 Department of Medical Oncology, Barts Cancer Centre, St Bartholomew’s Hospital, Barts Heath NHS Trust, London EC1A 7BE, UK; 4 Department of Medical Oncology, Mount Vernon Cancer Centre, East and North Hertfordshire Trust, London HA6 2RN, UK; 5 Health Systems and Treatment Optimisation Network, European Cancer Organisation, 1040 Brussels, Belgium; 6 Oncology Council, Royal Society of Medicine, London W1G 0AE, UK; 7 Kent and Canterbury Hospital, Canterbury CT1 3NG, UK; vpapadoster@gmail.com; 8 Faculty of Life Sciences & Medicine, School of Cancer & Pharmaceutical Sciences, King’s College London, Strand Campus, London WC2R 2LS, UK; 9 Faculty of Medicine, Health, and Social Care, Canterbury Christ Church University, Canterbury CT2 7PB, UK; 10 AELIA Organization, 9th Km Thessaloniki—Thermi, 57001 Thessaloniki, Greece

**Keywords:** artificial intelligence, oncology, machine learning, deep learning, natural language processing

## Abstract

The technological capability of artificial intelligence (AI) continues to advance with great strength. Recently, the release of large language models has taken the world by storm with concurrent excitement and concern. As a consequence of their impressive ability and versatility, their provide a potential opportunity for implementation in oncology. Areas of possible application include supporting clinical decision making, education, and contributing to cancer research. Despite the promises that these novel systems can offer, several limitations and barriers challenge their implementation. It is imperative that concerns, such as accountability, data inaccuracy, and data protection, are addressed prior to their integration in oncology. As the progression of artificial intelligence systems continues, new ethical and practical dilemmas will also be approached; thus, the evaluation of these limitations and concerns will be dynamic in nature. This review offers a comprehensive overview of the potential application of large language models in oncology, as well as concerns surrounding their implementation in cancer care.

## 1. Introduction

Artificial intelligence (AI) is a branch of computer science involved with creating machine systems that can mimic human intelligence and cognition. From a conceptual idea initially proposed by Alan Turing in the 1950s, the progression and advancement of AI have continued with great momentum [1,2]. The emergence of diverse AI subfields has since been embraced, including machine learning (ML), deep learning (DL), natural language processing (NLP), and computer vision [3].

AI’s revolutionary impact is noted in a spectrum of fields in all aspects of daily life, including healthcare and medicine, despite the attached strong historical dichotomy between its proponents and critics. Schwartz et al. notably highlighted in the New England Journal of Medicine that physicians may be wondering why the AI revolution in medicine has not yet occurred [4]. This is even more poignant and supportive of the long-anticipated disruptive eventuality of AI’s role in healthcare, given that this was published in the 1980s [4]. Medicine has previously experienced ‘AI winters’, where narratives of observers and stakeholders on the transformative role of new AI technology have been previously identified with inflated expectations incongruent with realistic outcomes, thus leading to reduced technological adoption [5]. 

As of late, novel advances in DL models have gained widespread public prominence and, importantly, new calls for optimism regarding AI systems [6]. AI’s remarkable success has been noted broadly in the medical field in disease diagnosis, treatment, and prognosis. A few examples notably include the analysis of medical imaging, extending into the interpretation of ECGs, pathological slides, ophthalmic images, and dermatological conditions, as well as its application in surgery with preoperative planning, intraoperative guidance, and surgical robotics [7,8].

Large language models (LLMs), which utilise DL and NLP, have taken the public and scientific community by storm, with consequent reinvigoration of discussions surrounding the role of AI in medicine [9]. Examples of LLM systems available on public domains include ChatGPT (Chat Generative Pre-Trained Transformer), Google BARD, Anthropic Claude, and Perplexity [10,11,12,13].

Oncology is not an exception to the changing landscape of AI and medicine. Oncology is entering a new age where the interplay and role of AI are no longer a theoretical possibility but a reality, with its approval for use in diverse clinical scenarios from cancer diagnostics and computer vision, including tumour detection in medical imaging and digital histopathology, to anticancer drug development and discovery with AI-driven target identification [14,15,16]. The versatility of LLMs’ function and application provides a potential opportunity for implementation in cancer care. Diverse examples of their possible application in oncology includes the extraction of data from electronic health records and reviewing next-generation sequencing (NGS) biomarker profiles to produce specific recommendations in personalised anticancer treatment [17,18]. It goes without saying that concurrent appreciation of pitfalls and challenges when considering future implementation is also essential. 

Given the novel advancement of LLMs coupled with their applicability for implementation in cancer care, this article aims to provide an overview of the role of LLMs in oncology. This article also aims to discuss the potential role of LLMs in creating a positive revolutionary driving force in oncology, as well as the contrasting potential for their negative disruption.

## 2. Methods

Medline/PubMed, CINAHL, Cochrane Library, EMBASE EMCARE, Trip Pro, Knowledge and Library Hub, Google Scholar, NIHR, and NICE Guidelines were searched from inception until January 2024 for publications in the English language reporting on LLMs, DL, and NLP. The search was carried out as follows:

✓ Neoplasms OR cancer OR Tumours/Tumors OR Oncology OR malignancies;
✓ Large language model OR LLM;
✓ GoogleBard OR ChatGPT OR Claude OR Perplexity.

The screening of the articles was performed manually by AC and WS based on the publication titles and abstracts. Of the articles retrieved, the reference lists of the relevant papers were checked to detect other articles that may be of interest for our review.

## 3. Large Language Model Function

**Figure 1:**
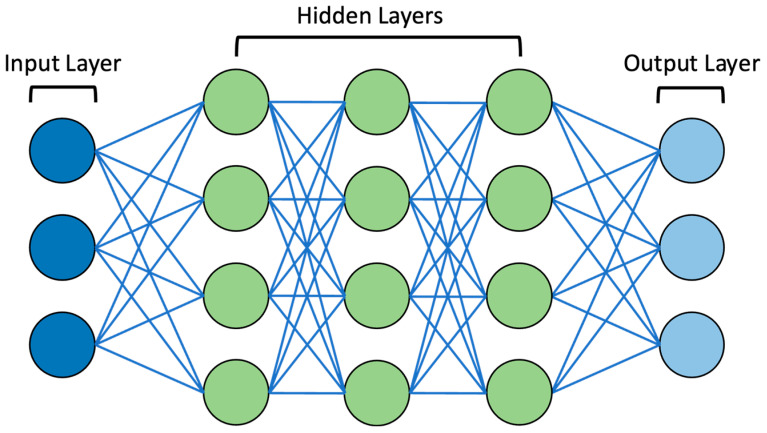
Diagram of example DL neural network. Note the presence of the input layer, ‘hidden’ layers (3 layers in this example), and output layer. These are connected by lines representing weighted connections.

ML systems use algorithms that can analyse and identify patterns in vast datasets. Furthermore, these systems can ‘learn’ from these data, thus recognising new data input and allowing for informed decision making, a dynamic process that is not fixed in nature [19]. With the increasing complexity of data due to their increasing size and the intricacies between data input and output, ML paved the way for the development of DL [3]. DL is based on multi-layer artificial neural networks (ANNs), which have the power to model arbitrarily complex associations, thus providing the capability to ‘learn’ these complex relationships alongside the ability for independent decision making [19]. ANNs were inspired by the architecture and function of the human brain, originating from attempts to create mathematical models in neurobiology and cognitive psychology [20]. McCulloch, Pitts, and Hebb notably first attempted to construct an abstract mathematical model of the nervous system in the late 1940s and early 1950s, utilising biological bases for neuronal modelling [20]. Subsequently, in mathematical models, neurons were termed ‘nodes’ or ‘artificial’ neurons. The classic graphical representation of ANNs involves an input layer and an output layer, which are linked by a series of interconnected ‘hidden’ layers comprising multiple ‘nodes’ [21]. As highlighted, one ‘node’ of ANNs represents a neuron, and each node connects to another via a weighted connection. Once the defined threshold is exceeded, that node is activated, which connects to other neurons at the next synaptic junction and so forth, eventually passing through multiple layers [21]. The interconnection patterns formed by the input layer, ‘hidden’ layers, and output layer are referred to as the network architecture [22]. It should be noted that ‘deep’ in DL references the depth of layers in the network architecture. If there are more than three layers in the ANN, including the input and output layer, it is considered to be a DL algorithm [19]. The architecture of an example DL algorithm can be seen in Figure 1.

Most ANNs are feed-forward, meaning the flows of weighted connections are unidirectional from input to output. Flow can also be back-propagated, thus identifying the error associated with each node and making it amenable to computational algorithmic change. Fundamental neural network methods include multilayer perceptrons, recurrent neural networks, and convolutional neural networks [23]. With the promise of precision oncology, use of ANNs has been proposed in a variety of oncological settings. Despite limited routine clinical use at present, some models have been approved by the FDA and adopted into the clinical environment. For example, convolutional neural networks have been used to stratify indeterminant pulmonary nodules identified through CT imaging, in addition to using digital histopathology to predict breast and prostate cancer diagnoses [15,24,25].

NLP enables computers to process the human language using computational linguistics combined with ML and DL algorithms [26]. Applications of DL to NLP and breakthroughs in generative AI paved the way for LLMs, which utilise DL models that generate outputs when prompted, having analysed the raw data [27,28]. LLMs are typically based on transformer architecture, which is a type of network architecture first proposed by Vaswani et al. in 2017 [29]. Subsequently, LLMs began to emerge in 2018, with their capability and number of analysed parameters advancing at extraordinary rates [30]. They comprise multiple layers of ANNs, each with an extensive number of parameters, which can be fine-tuned during the training process with unlabelled text from large datasets [27]. Another layer of ANNs known as the attention mechanism can be added to further enhance the fine-tuning process [31]. Based on the complex human cognitive function of attention, attention mechanisms are able to focus on specific parts of datasets and place increased weighting on certain elements depending on input data [29].

Through training with huge datasets, LLMs are able to form appropriate responses when prompted. Zero-shot and self-supervised learning methods are used to facilitate the correct use of grammar, semantics, and conceptual relationships. Thus, through the training process, LLMs are able to predict subsequent words in a sentence depending on relevance and patterns acquired [31]. 

**Figure 2:**
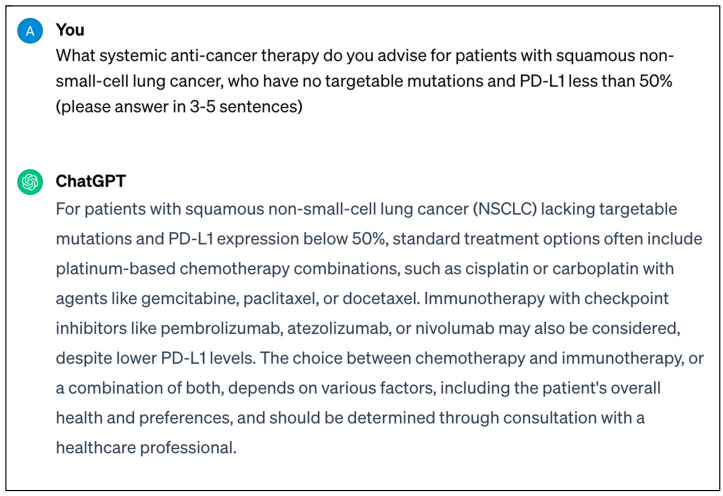
Screenshot of real-time response from ChatGPT-3.5 regarding systemic anticancer therapy that can be utilised for patients with squamous non-small-cell lung cancer.

An example highlighted earlier includes ChatGPT, which, following its release towards the end of 2022, remains one of the most well-known LLMs to date, taking the world by storm with concurrent excitement and concern after its availability in the public domain [10]. Its most recent release, GPT-4, has over 100 trillion parameters, as well as the ability to process text and image input, which is superior to GPT-3.5. An example text prompt and response from ChatGPT can be seen in Figure 2. 

Most notably, LLMs can generate human-like, patient-friendly responses when prompted and remember data input earlier within conversations, which can facilitate communication with AI systems in a human-like manner. Consequently, it is unsurprising that LLMs have re-sparked the debate of whether AI systems truly understand natural language and hence appreciate both the physical and social scenarios that language can describe [32]. Some argue that LLMs can understand language and thus perform general reasoning, albeit at present not at the level of humans. However, others state the impossibility of LLMs understanding language, as they have no experience of the world and their training is guided by statistical algorithms, which teach the form of language rather than the true meaning [33]. This complex debate will go further than academia, as the level of true machine understanding will influence our level of trust and determine the spectrum of autonomy in its application in oncology and beyond.

## 4. A Cause for Revolution

LLMs have the potential to be incorporated into a wide variety of settings in oncology. They can be harnessed throughout the oncology patient’s journey, from symptom onset and evaluation to survivorship or disease progression.

### 4.1. Oncological Clinical Practice

Cancer diagnostic workup is complex, requiring comprehensive medical history taking, physical examination, as well as analysis of blood tests, histopathologic morphology, algorithmic immunohistochemistry, and various forms of radiological imaging. LLMs can support these processes.

LLMs have shown promise in the analysis of laboratory medicine test results as well as improving the accuracy and efficiency of radiology image diagnoses in real-time, facilitating swift interpretation [34,35]. From a radiological perspective, in the context of cancer diagnosis or exclusion, the role of LLMs can also extend into supporting cancer screening services. Feasibility of using LLMs for the analysis of breast cancer screening mammograms has been demonstrated, which may eventually improve clinical workflow, alongside supporting the radiological decision-making process [36]. 

Furthermore, extraction of data from medical records and previous radiological imaging can be supported by LLMs. This is a valuable tool in medicine, which can prove to be especially useful in oncology, where a patient’s treatment may span several years and require multiple lines of anticancer therapy with sequential interventions [17,18]. Critical parameters for diagnosis and management can be filtered from vast datasets in a form that is clear and concise, thus ensuring all crucial clinical information is available to support the patient’s treating oncologist. Additionally, LLMs can support oncologists with documentation and administrative duties. Although essential, these requirements have been noted to consume approximately 25% of physicians’ workload [37]. Through the conversion of unstructured notes to structured formats and the creation of standardised reports, LLMs can ease administrative duties in routine cancer care or clinical trials [38]. Also, the integration of voice-to-text technology and LLMs can support the introduction of automated dictation and prompt-triggered chart review [38]. As healthcare organisations are transitioning from paper to electronic health records, the opportunity to integrate LLMs into these systems will arrive. Thus, this will present the potential to reduce oncologists’ administrative burden as well as ameliorate diagnostic accuracy, treatment planning, and outcomes by supporting the process of distilling large quantities of stored patient data [39]. 

Tissue diagnosis remains key to conclusively establishing the presence of malignancy and thus guides oncological decision making. From a clinical pathology perspective, LLMs can support the pathologist with immunohistochemistry stain sensitivities, tumour grading, as well as the formation of initial differential diagnoses [40]. Additionally, LLMs can support the interpretation and summarisation of these reports for oncologists with increased weighting on pertinent areas through the use of attention mechanisms. 

Support in the clinical decision-making process can also be provided to oncologists by LLMs, which can play the role of a ‘virtual assistant’ [27]. Multiple studies have assessed the ability of LLMs as a decision support tool for answering questions regarding the treatment and management of various malignancies [41,42,43]. Notably, Sorin et al. used ChatGPT in order to evaluate the potential use of LLMs as a support tool in the breast tumour board, a multi-disciplinary meeting where specialists from different backgrounds discuss the management of complex breast cancer cases [41]. Ten real-world cases were assessed by the tumour board and ChatGPT, where clinical recommendations made by ChatGPT were concluded to be in line with 70% of the cases discussed by the tumour board. Additionally, when prompted, the LLM was able to provide concise case summaries and clinical reasoning for its conclusions [41].

Similarly, Haemmerli et al. evaluated the role of ChatGPT in their institution’s central nervous system tumour board for glioma adjuvant therapy decision making. The gold standard tumour board decisions, supported by evidence-based medicine and consensus of the multidisciplinary team, were compared to outputs provided by ChatGPT [42]. The LLM was able to provide good treatment recommendations and therapy regimens, with overall moderate agreement with the tumour board’s decisions. However, it was noted that there was poor performance and limited precision in the diagnosis of specific glioma subtypes [42]. Another observational study assessed the capacity of ChatGPT to advise on guideline-based systemic treatment regimens for newly diagnosed advanced solid tumours. In the 51 distinct diagnoses that were assessed, ChatGPT evidenced the ability to identify suitable cytotoxic chemotherapy, targeted therapy, and immunotherapy agents in accordance with the National Cancer Comprehensive Network (NCCN) guidelines [43]. Given this ability of LLMs in clinical decision making and recommendations for systemic anticancer therapy regimens, it remains unsurprising that the use of LLMs in clinical trials has commenced. In a first-of-its-kind, randomised, single-blinded, parallel assignment clinical trial, the primary outcome measure of the investigators will be to establish the influence of LLMs on treatment plans for patients with gastrointestinal malignancies [44]. 

One can also consider the role of LLMs in analysing NGS panels in precision oncology. NGS panels are increasingly utilised in guiding treatment for patients with advanced cancers in order to identify actionable mutations associated with specific targeted therapies and immune-based therapies. However, there is evidence that this is often underperformed and underutilised by oncologists in the community setting [45]. Additionally, the trajectory of molecular testing and consequent prescribing patterns have not shown distinct improvements with time [46]. Through the identification of clinically relevant biomarkers, LLMs can be used in evidence-based interpretations of NGS panels and consequently provide recommendations for treatment [17,18]. By alleviating the challenges in the interpretation of test results, LLMs can provide systemic support to oncologists by reducing disparities and providing optimal care in the age of precision oncology [47].

### 4.2. Cancer Patient Support and Education

LLMs can be considered ‘virtual assistants’ not only for oncologists but also for cancer patients. LLMs have the potential to support patient disease understanding and engagement through the delivery of medical information in real time, which can be provided in a concise and patient-centred approach [48]. Despite controversy surrounding the public accessing medical information online, it is important to appreciate the frequent use of the internet for health-related purposes at present [49]. Not soon after the release of ChatGPT, it was shown to be capable of providing responses to common cancer misconceptions that are accurate and similar to answers provided by the National Cancer Institute’s (NCI) ‘Common Cancer Myths and Misconceptions’ web page [50]. 

Several further studies evaluating the role of LLMs in answering cancer patients’ common questions have since been completed [51,52,53]. Haver et al. were able to highlight ChatGPT’s ability to provide appropriate answers in 88% of the 25 questions it was asked regarding breast cancer prevention and screening [51]. Yeo et al. similarly investigated ChatGPT’s performance in answering questions about liver cirrhosis and hepatocellular carcinoma management as well as emotional support. They highlight a greater proportion of accurate responses about basic knowledge, lifestyle, and treatment domains when compared to responses related to diagnosis and preventive medicine [52]. Notably, for caregivers of patients with newly diagnosed hepatocellular carcinoma, ChatGPT was able to give multifaceted psychological and practical advice [52]. Other LLMs, such as Perplexity, Bing AI, and Chatsonic, have also evidenced the production of generally accurate responses to common cancer-related queries [53].

### 4.3. Educating Students and Healthcare Professionals in Oncology

In addition to cancer patient support and education, the application of LLMs as an education tool can also be considered for healthcare professionals and students in oncology. Educational benefits can be achieved with LLMs through diverse methods to enhance the learning experience. This includes creating content to facilitate the learning process, including the generation of realistic oncology clinical vignettes, customisable simulated clinical cases providing immediate feedback, and fast access to information through the summarisation of the medical literature [54]. In the medical education setting, AI systems have been previously identified as supporting and providing a personalised learning experience [55]. With their responsible use, LLMs can promote the personalised learning model in the context of oncology and beyond through individualised feedback as well as by breaking down complex and multifaceted concepts in cancer care and evidence-based treatment strategies [38]. The integration of LLMs and the gamification process also provides another exciting outlook on future oncology education models in simulated and non-simulated settings, with broad potential improvements in learning retention and skill acquisition [56].

### 4.4. Oncology Research

Given the vast number of parameters that LLMs are trained with, coupled with the real-time ability of data extraction, summarisation, and text generation, LLMs can be harnessed to support the progression of oncology research. Their utility can be considered from a research process and academic writing perspective. Firstly, LLMs can support the completion of comprehensive literature reviews [48]. Through their appropriate use in evidence synthesis and data extraction, they could also facilitate automatization in the conducting of narrative review synthesis for systematic reviews [57]. Furthermore, LLMs have shown great potential in generating high-precision queries in systematic reviews [58]. 

The data extraction ability of LLMs can also be enhanced through fine-tuning. This includes pre-trained LLMs in the generative and discriminative setting, i.e., they can generate responses to a question when prompted in a given context and classify input data into predefined labels [59]. Domain-specific LLMs, such as BioMedLM and BioGPT, are trained with data from the biomedical literature on PubMed and can be fine-tuned with gold standard oncology corpora [60,61]. Thus, this will facilitate the ability of LLMs to yield high-quality results for extraction tasks in the oncology domain. The release of LLMs with the option of customisable models provided by the community will also likely accelerate the process of tailored solutions and addressing oncology-domain-specific queries [62].

Data analysis can also be supported with the generation of codes for visual data presentation, in addition to coding that can be input into statistical software systems, such as python version 3.8.5, R version 4.0.2 (2020-06-22), or Stata 7SE [57]. Notably, OpenAI has introduced an ‘advanced data analysis’ feature available on GPT-4.0, which can further eliminate barriers that researchers may face with data analysis [10]. The model can support a variety of data and programme files. In addition to performing statistical analysis when prompted, corresponding python code is also provided, allowing for reproducible data analysis. Thus, appropriate oversight can be maintained, and coding can be modified as required to improve data output. Suggestions are also offered for options for further data manipulation. Easy access to such powerful AI tools in oncology research can dismantle barriers researchers may face in addition to improving the efficiency of data manipulation, thus facilitating further cancer data exploration, coding, and tackling empirical problems in oncology.

Assistance in the writing process can be provided by LLMs, which can be efficacious in improving the communication of ideas and results [54]. This can be especially useful for non-native-English-speaking researchers, and it can subsequently improve equity and inclusivity in research [54]. 

Overall, LLMs can complement traditional research methodology. They have the potential to act as a catalyst in the already rapidly evolving and exciting domain of oncology research and contribute to the acceleration of knowledge acquisition to improve cancer care [63].

## 5. A Cause for Concern

LLMs have incredible potential to revolutionise modern-day oncology. Nevertheless, several limitations and major challenges must first be overcome in order to facilitate the integration of LLMs into oncological practice. 

### 5.1. Data Accuracy 

Despite the identified impressive ability of LLMs to answer prompts pertaining to oncology, it is important to note that LLMs carry a risk of providing false responses, which are known as ‘hallucinations’ [9]. Through the process of AI hallucinations, LLMs perceive patterns that are fictitious or imperceptible to the human observer, with the consequent outputs being nonsensical or completely incorrect [64]. Publications evaluating the role of LLMs in cancer care also indicate that incorrect or suboptimal outputs are not infrequent, which can be noted in the aforementioned studies. Thus, concerns remain around the reliance on and provision of contradictory or false information provided by LLMs, which could negatively impact management and, subsequently, patient outcomes [2,65]. It goes without saying when considering the automation of healthcare information and counselling provision by LLMs that sufficient oversight must be in place in order to prevent dissemination of incorrect medical information that may be harmful to patients. 

It should be noted that different strategies exist to overcome LLM hallucinations, which can be separated into two categories, data-related methods or modelling and interference methods [66]. Data-related methods include ensuring that high-quality cancer data are used for pre-training LLMs. Fine-tuning can also be utilised by adapting the LLM to oncology-specific domains [67]. Retrieval augmented generation is a framework that can further reduce the risk of hallucinations by grounding LLMs with knowledge from external reference textual databases [68]. Modelling and interference methods include reinforcement learning from human feedback, which involves a human evaluator ranking LLM output efficiency [69]. Appropriate prompt strategies, notably chain-of-thought prompting, which uses a stepwise approach and aggregates LLM output, can reduce incorrect responses by encouraging LLMs to reason prior to answer arrival [70]. The sampling temperature of LLMs, which guides the ‘creativity’ of output, can also be adjusted. It is a scalar value from 0.0 to 1.0 and adjusts the probability distribution of subsequent word selection in LLM output. The higher the temperature, the more random and ‘creative’ the output will be. On the contrary, lower temperatures will result in more deterministic output and hence more repetitive and focussed outputs in line with patterns from cancer training data [71]. It goes without saying that when used in the oncological clinical setting, appropriate temperatures for optimal LLM output will need to be established. Additionally, a variety of methods will need to be harnessed to reduce and avoid hallucinations when LLMs are used in the oncology domain. Also, it is important to consider that LLMs provide responses based on the datasets that they were trained on; these can include large collections of textual information from books, articles, and websites [41]. Consequently, for future implementation into oncological practice, datasets used for training must be up to date so that evidence-based responses can be generated, including, for example, when utilised as a clinical decision support tool for oncologists or as a virtual assistant for cancer patients. Of note, ChatGPT-3.5 is trained with data that are limited to January 2021 [10]. As a result, new advances in oncology, including novel research developments and best practice guidance updates, would not be incorporated into the LLM’s response outputs, which is especially concerning given the fast-advancing nature of oncology research [42]. An additional limitation to the integration of LLMs in oncology is the need for diverse and inclusive datasets that can be used as training data [14]. It is imperative that AI algorithms are expanded to include equity, diversity, and inclusion concepts, with training datasets reflecting the true patient population [72]. Otherwise, there is a risk of discrimination alongside the automation and propagation of existing biases, which may lead to responses that are inaccurate and potentially harmful to patients [73]. The challenges in ensuring that LLM training sets and AI algorithms are diverse and inclusive can be considered similar to that of the application of clinical trial results, where complex multilevel barriers exist in ensuring that a diverse population set of patients with cancer is enrolled [74].

In order to mitigate concerns regarding the accuracy of data output and positively influence LLM performance in the oncology setting, prompt engineering can be leveraged, which is a new field of research involved in the development and refinement of prompt words to optimise LLM output [75]. Thus, prompt engineering will be an important emerging skill for users of LLMs, including patients and oncologists alike. Different styles and types of prompts can be utilised. For example, in zero-shot prompts, the LLM is expected to perform a task it has not been specifically trained on, and hence without exposure to previous examples [76]. Few-shot prompts involve task completion where the LLM has previously only been exposed to a few initial examples; thus, the task is completed with appropriate generalisation to unseen examples [77]. Notably, Singhal et al. were able to demonstrate the effectiveness of prompt engineering strategies by improving the output accuracy of the LLM Flan-PaLM in answering USMLE-style questions through chain-of-thought, few-shot, and self-consistency prompting strategies [78]. Overall, adequately engineered prompts will be key to maximising the performance of LLMs as well as reducing unsatisfactory responses in the oncological setting. In practice, however, challenges remain in the application of prompt engineering. These include prompt robustness and transferability [79]. Thus, when used in the oncology domain, patients and oncologists may receive different responses even if the same prompt framework is used [80]. Additionally, given that prompt engineering performance is dependent on the inherent capabilities of individual LLMs, prompt strategies deemed effective for one LLM may not be appropriate for another [80]. Appropriate guidance will need to be developed in order to ensure appropriate prompt strategies are used to guide LLM output for various tasks in the oncology domain. It will also be important for oncologists and patients to be involved in the development of human evaluation frameworks and LLM response evaluation frameworks, thus supporting researchers to measure progress and identify and mitigate potential harm [78].

### 5.2. Accountability

Oncological decision making and treatment planning are multimodal; a patient-centred approach and evidence-based practice are key to providing the highest quality of care. However, prompts from LLMs often show a lack of accountability for the subtleties of cancer care, such as co-morbidities, previous lines of treatment, and, vitally, patient values and treatment goals [43]. The accountability and responsibility of AI systems in medicine have long been key ethical concerns and limitations to broader implementation due to the gravity of the consequences that may arise when mistakes are made [81]. The European Commission and the US Food and Drug Administration (US FDA) have released policy proposals and guidance for the use of AI systems as well as the use of clinical decision support tools [82,83]. However, at present, there is still a lack of comprehensive legislation adequately protecting the fundamental rights of patients surrounding the use of AI-driven clinical practice [14]. In recent years, the concept of ‘meaningful human control’ has been increasingly referred to in the context of automated systems, which is the idea that humans should ultimately have control over computers and, consequently, moral responsibility for decisions made [84]. The levels of automation of LLMs in oncology can potentially range from providing contextual information as a clinical support tool to the direct management of oncological conditions without oversight. Thus, it will be key for relevant stakeholders to address future frameworks to integrate the concept of meaningful human control alongside comprehensive legislation in order to ensure the ethical use of automated systems, such as LLMs, in oncological practice and beyond [85].

### 5.3. Data Security

Another key ethical limitation of the integration of LLMs into oncology practice is concern regarding data security and the protection of patient confidentiality. At present, LLMs are not compliant with the US Health Insurance Portability and Accountability Act of 1966, a federal law that serves to protect sensitive patient data from being shared without patient consent [17,18,86]. Thus, there will be a risk of data breach if patient data are input when LLMs are utilised to support or provide patient-centred and evidence-based cancer care. This will remain a major limitation in LLM implementation as oncological practice shifts further into precision and personalised care for cancer treatment and thus requires further specific and sensitive patient information.

Notably, in the United Kingdom, the National Cyber Security Centre advises caution regarding the data that are submitted to LLMs for prompts, as input data can be visible to the organisation providing the LLM [87]. Similarly, concerns in Europe have led to the formation of a task force on ChatGPT by the European Data Protection Board [88]. As a result, not only is there a risk individual data breach that can be accessed by LLM providers, but also breaches secondary to adversarial cyber-attacks that have the capability of exploiting AI infrastructures, leading to compromise and manipulation of patient data. Undoubtably, for the future implementation of LLMs in oncological practice and healthcare, data protection concerns must be appropriately addressed.

### 5.4. Research Integrity

Despite the promising contributions that LLMs can offer in supporting oncology research, barriers and concerns exist regarding their application in the scientific process. Firstly, issues regarding plagiarism and author misrepresentation can be considered [30]. As highlighted, LLMs are capable of providing responses to scientific prompts; however, these are typically without appropriate citation from the original source [63]. Thus, researchers are at risk of plagiarism, as well as being susceptible to AI hallucinations, biases, and the limited transparency of the provided data. Limited LLM transparency in response generation from input queries, model architecture, and algorithms also contribute to so-called ‘black box’ issues, making interpretability and the decision-making processes a challenge [77]. A level of human verification or fact-checking will be imperative to prevent the dissemination of inaccurate research if LLMs are used in this process [63]. At present, the unacknowledged use of research can be identified through anti-plagiarism software; however, as LLMs evolve, there is a risk that this may be circumvented. Thus, referencing issues and risk of academic fraud remain key concerns [54]. AI-generated text detection tools are being developed; however, initial studies highlight the challenges in differentiating LLM-generated text versus non-LLM-generated text in practical scenarios [89].

Use of LLMs as an information source for research also raises concerns regarding the negative impact on critical thinking, which is achieved through the mental process of discernment, analysis, and evaluation to arrive at a logical conclusion [90]. Through their inappropriate use, LLMs can bypass these processes, which risks the externalisation of factual knowledge as well as the foundations of oncological reasoning, which has implications beyond the maintenance of research integrity [38].

*Nature* notably defined its policy on the use of LLMs in scientific publications in the beginning of 2023. It was highlighted that LLMs cannot be credited as an author, as they do not carry responsibility or accountability for their work. Additionally, it was noted that the use of LLMs should be documented in the methods or acknowledgement sections of publications [91]. Other journals have also promptly released guidance on the use of LLMs in scientific manuscripts [92,93]. Policies will need to evolve concurrently with LLMs with close cooperation and supervision by the scientific community alongside AI ethics and safety experts to ensure that LLMs do not compromise but rather enhance the rigor, transparency, and reproducibility of research [30]. Overall, the maintenance of academic and research integrity in oncological research will be pivotal in advancing our knowledge base and providing the best care for future patients.

## 6. Strengths and Limitations

This review serves as a foundation for discussion as we highlight the potential roles of LLMs in oncology, as well as concerns and barriers regarding their future implementation. We capture the excitement of their prospective application and the contrasting associated gravity of concerns. A key limitation to this review includes the infancy of LLMs; despite a recent surge in publications concerning the use of LLMs in oncology, their overall application in the literature remains low. Additionally, the capabilities of LLMs are fast-evolving alongside the ethics surrounding their use in cancer care, limiting the ability to draw conclusions regarding their potential use in oncology.

## 7. Conclusions and Future Directions

The progression and advancement of AI systems and LLMs are inevitable. As the integration of AI in cancer care continues, the prospective application of LLMs in oncology fosters great promises. The versatility of LLMs is impressive, facilitating their potential utilisation in both oncological practice and research. However, it is of the utmost importance to consider the limitations and risks associated with their use. It goes without saying that the foundations of evidence-based practice, patient-centred care, and scientific research should not be compromised in attempting to prematurely introduce AI systems into oncology. Key stakeholders, including policy makers, oncologists, AI ethics experts, and the wider multi-disciplinary team, will need to address these concerns in order to allow for effective and safe implementation of the use this technology. As AI systems advance, new ethical and moral dilemmas will come to light. Thus, the appreciation of concerns and ethical issues regarding the use of LLMs in cancer care will not be a static process but rather one that is dynamic and concurrently advancing. It will be our collective responsibility to ensure that AI systems are used at the highest of standards to ensure best practice and the highest quality of care delivery to cancer patients, whilst adhering to the fundamental principles of ethics.
